# Editorial: Overcoming boundaries in public health: Advances in international and global health

**DOI:** 10.3389/fpubh.2022.1044157

**Published:** 2022-10-14

**Authors:** Ippazio C. Antonazzo, Janet Sultana, Pietro Ferrara

**Affiliations:** ^1^Center for Public Health Research, University of Milan—Bicocca, Milan, Italy; ^2^Exeter College of Medicine and Health, Exeter, United Kingdom; ^3^Health Direction, Istituto Auxologico Italiano Research Hospital-IRCCS, Milan, Italy; ^4^Laboratory of Public Health, IRCCS Istituto Auxologico Italiano, Milan, Italy

**Keywords:** boundaries of diseases, global health, infection disease epidemiology, international health, burden of disease

Global health has, as a rule, been considered as the participation of governments and international agencies to address health issues that transcend national boundaries and, thus, require responses at an international and a global level (1). “Global health” itself is a broad concept (2), encompassing—according to the Consortium of Universities for Global Health Executive Board—“notion (the current state of global health), an objective (a world of healthy people), or a mix of scholarship, research, and practice (with many questions, issues, skills, and competencies)” (3).

The present Research Topic focuses on *Overcoming Boundaries in Public Health: Advances in International and Global Health*, contexts where it is always a challenge to design and implement solutions for overcoming boundaries of diseases. The World Health Organization (WHO) has suggested the implementation of several goals to strengthen global health response: supporting national surveillance and response systems, fostering global partnership, improving health safety in travel and transport, sustaining human rights, and focusing research efforts.

To achieve these pillars, research represents the key to identify problems of current interest. In this sense, this Research Topic collected important contributions to these evolving challenges.

People move and, along with this movement of people, diseases can start in one area and quickly reach another part of the world (4). Travel, in particular, is an independent driver of the epidemiology of infectious disease (5). The special issue paper by Du et al. highlights an ecological correlation between international travel and new human immunodeficiency virus (HIV) infections. While further research is needed to better exploit this relationship, authors called for control and preventive measures, including health promotion initiatives targeted to travelers and policy interventions to address the positive medium/long-term potential of travel-related HIV epidemiology, such as additional prevention tool for the reduction of HIV transmission in high-risk population (6).

Tuberculosis is another topic of great interest in global health, also being the first infectious disease declared as a global health emergency worldwide (7, 8). According to WHO, 1.5 million people die from tuberculosis each year, being the top global infectious killer (8). Early diagnosis and treatment are crucial to prevent tuberculosis transmission and control its global impact. In this regard, Hu et al. conducted research to investigate the value and feasibility of early diagnosis of tracheobronchial tuberculosis by actively performing bronchoscopy in patients with pulmonary disease, which correlates with less risk of tracheobronchial stenosis and improves the prognosis.

The severe acute respiratory syndrome coronavirus 2 (SARS-CoV-2) spread wreak havoc on human societies over the last two years. The health burden of the coronavirus disease 2019 (COVID-19)—the disease associated with SARS-CoV-2—overcomes the direct consequences of the infection. The restrictive measures imposed by governments and authorities to stop viral transmission and prevent COVID-19 cases, in fact, affect people's health and, above all, have had an important impact on the mental health of people (9). Indeed, the COVID-19 crisis is characterized by extreme uncertainty, which caused mental health problems, psychological distress, and protective behaviors, as highlighted in the study by Chung et al. The article provides an interesting insight into the Hong Kong experience, where the great level of fear owing to COVID-19 was accompanied by less severe psychosocial distress in the general population compared with other groups in Taiwan. Reasons for this difference might date back to the 2003 SARS epidemic, which would lead to better pandemic preparedness at government and community levels, including awareness of infection control and resilience against COVID-19 distress in the general public.

The global burden of infectious diseases is particularly high in the African region, where some conditions—among which HIV, malaria, and tuberculosis—cause morbidity, mortality, and economic inequality compared with the rest of the world (10). In this sense, context-specific epidemiological research is key to identify relevant elements to inform public health interventions. However, Perryman et al. pointed out that several gaps exist in regional research outputs, with African researchers pressured by systemic barriers, including the low number of high degree scholars, difficulties in accessing research infrastructure, and scarce funding opportunities. Authors, therefore, submitted a scoping review protocol to describe methods in which they intended to investigate the changing patterns of first and last authorship in African research on infectious diseases, with the ultimate goal of identifying relevant gaps in local authorship and high-income country collaborations.

In a nutshell, public health efforts to address health issues that transcend national boundaries move from the complete knowledge of all the aspects of diseases to the identification of the most appropriate actions to contain them, to the implementation and evaluation of global interventions. This Research Topic documents real, practical experiences that enrich the current body of evidence in global health, collecting important but still poorly understood pieces of research surrounding this topic, spanning from epidemiology to public health implications and responses.

Finally, the Special Issue *Overcoming Boundaries in Public Health: Advances in International and Global Health* contributes both to advance and stimulate the scientific debate about those diseases that do not end at international borders.

## Author contributions

PF wrote the editorial. IA and JS participated in reviewing and editing the text. All authors have read and agreed to the published version of the manuscript.

## Conflict of interest

The authors declare that the research was conducted in the absence of any commercial or financial relationships that could be construed as a potential conflict of interest.

## Publisher's note

All claims expressed in this article are solely those of the authors and do not necessarily represent those of their affiliated organizations, or those of the publisher, the editors and the reviewers. Any product that may be evaluated in this article, or claim that may be made by its manufacturer, is not guaranteed or endorsed by the publisher.
